# Primary healthcare professionals’ attitudes toward patients with current or previous drug use

**DOI:** 10.1080/02813432.2024.2349063

**Published:** 2024-05-09

**Authors:** Lars Garpenhag, Disa Dahlman

**Affiliations:** a Department of Clinical Sciences Malmö, Center for Primary Health Care Research, Lund University/Region Skåne, Malmö, Sweden; b Department of Clinical Sciences Lund, Psychiatry, Faculty of Medicine, Lund University, Lund, Sweden; c Malmö Addiction Center, Region Skåne, Malmö, Sweden

**Keywords:** Attitude of health personnel, primary healthcare, drug users, opioid assisted treatment, health equity

## Abstract

**Objective:**

People with current or previous drug use (PCPDU) often lack long-term healthcare contacts in primary healthcare (PHC). While international research has shown negative attitudes toward PCPDU in healthcare, PHC professionals’ attitudes toward PCPDU have not been assessed in Sweden. The aim of this study was to investigate PHC professionals’ attitudes to PCPDU, and to compare attitudes toward people who actively use illicit drugs with those toward patients in opioid assisted treatment (OAT).

**Design:**

In this survey study, respondents were asked for background data, and their attitudes toward patients using illicit drugs, OAT patients and patients with depression were assessed by using an adapted version of the Medical Condition Regard Scale (MCRS).

**Setting and subjects:**

Nurses and physicians at primary healthcare centers (PHCCs) in Skåne, Sweden.

**Main outcome measures:**

Mean MCRS scores, dichotomized responses to MCRS items, and associations between MCRS score and background covariates (age, sex, profession and duration of professional experience).

**Results:**

Eighty-nine PHC professionals from 13 PHCCs responded (approximately 39% of relevant workforce). The median MCRS score was 44 for patients with illicit drug use and patients in OAT, and 51 for patients with depression. Drug use and OAT displayed similar minimum, maximum and interquartile range values as well, while scores regarding depression displayed a higher minimum value and smaller spread. No significant associations were found between background covariates and MCRS scores for either drug use or OAT.

**Conclusions:**

The results indicate widespread negative attitudes to PCPDU, with implications for health equity in the clinic. Further studies are needed to see if the results reflect attitudes in Swedish PHC in general.

**Key Points**
- People with current or previous drug use (PCPDU) often lack necessary primary healthcare (PHC) and are commonly subject to prejudice.
- Swedish PHC professionals held more negative attitudes toward PCPDU than toward patients with depression.
- Attitudes toward patients with active drug use and patients in opioid assisted treatment (OAT) were almost identical.
- Study findings have potential implications for the health of PCPDU as well as health equity in the clinic.
- Widespread negative attitudes to PCPDU in our sample indicate the need of larger-scale studies of attitudes toward PCPDU in Swedish PHC.

## Introduction

People with current or previous drug use (PCPDU) – including patients in opioid assisted treatment (OAT) – are at increased risk of a range of negative health outcomes and generally have significant healthcare needs that are not met by the healthcare system [1–5]. Several studies have stressed that people who use drugs and patients in OAT lack long-term healthcare contacts in primary and outpatient care, necessary for effective treatment and prevention of chronic somatic conditions [6]. Since primary healthcare (PHC) services serve important purposes not only as providers of healthcare, but also as entry points to the rest of the healthcare system, deficient interactions can have significant consequences, especially for vulnerable populations [7]. Besides being a way to avoid unnecessary human suffering, regular PHC contacts have been shown to be associated with decreased levels of hospitalization of users of illicit drugs [8].

One particular problem that PCPDU face is a risk for poor treatment in healthcare settings due to the stigma associated with drug dependence [9–11]. A substantial body of research has shown that internationally, negative attitudes toward people who use drugs are common among healthcare professionals and students, and that attitudes are consistently and substantially more negative than those toward most other patient groups [12–15]. Several studies have found poor attitudes toward patients who use drugs in PHC services [16,17], and with few exceptions [18] attitudes have been found to be more negative in PHC than in other branches of healthcare [13,19,20]. Importantly, the stigma associated with illicit drug use does not necessarily disappear because a person stops actively using. Although a UK study showed that health professionals held less negative attitudes toward substance using patients who were in remission [14], a number of qualitative studies suggest that there is stigma associated with enrollment in OAT as well, experienced both in the dispensing setting and in contacts with other healthcare services [10,21,22].

A host of factors have been proposed to negatively influence health professionals’ attitudes to PCPDU, including beliefs about the controllability of substance use and the responsibility of the individual [15], the endorsement of conservative ideology and values [23] and insufficient training or knowledge [19,24]. Concerning PHC in particular, a notion among professionals that such services make an inappropriate setting for PCPDU patients has been observed as well [16]. Meanwhile, treatment optimism [20] and organizational support [15,25] have been found to be associated with more positive attitudes. Professional experience has been attributed a somewhat ambiguous role in this respect. While general professional experience has been shown to be associated with negative attitudes [13], greater exposure to patients who use drugs in particular is associated with more positive attitudes [20,24,26]. According to several studies, male professionals and students display more negative attitudes to substance using patients than their female colleagues, a fact that has brought attention to the possible influence of gender-related factors as well [19,27].

Negative attitudes are in turn known to affect the quality of healthcare [15,24], as well as access to services [11,17,18]. In addition, anticipations of stigmatizing treatment can have unfortunate effects on healthcare seeking behaviors among PCPDU, for instance, making them postpone or outright avoid care [9,11].

Despite the potentially detrimental consequences that negative attitudes can have on healthcare delivery and patient health, PHC professionals’ attitudes toward PCPDU have not been assessed in Sweden. Sweden makes an interesting setting because it displays an unusual combination of having a traditionally restrictive narcotics policy and universal healthcare services, which include a comprehensive PHC system. In addition, to the best of our knowledge, there exist no previous studies that compare attitudes toward patients in OAT to those toward patients with active drug use. In this study, we aimed to investigate Swedish PHC professionals’ attitudes to PCPDU, and to compare attitudes toward people who use illicit drugs with those toward OAT patients.

## Materials and methods

### Setting

The study was conducted in primary healthcare centers (PHCCs) in the Skåne region (pop. 1.35 million) in southern Sweden. The Swedish healthcare system is comprehensive, mostly tax-financed, and universal. Consequently, health services delivered by providers tied to the public healthcare system are heavily subsidized to the individual patient. PHC is delivered mainly through PHCCs, normally by multidisciplinary teams. Even though they are not formally serving as gatekeepers, PHCCs are considered a first line of healthcare to which residents should turn with non-emergency health problems [28]. The quantity and exact scope of PHC services differ between different regions. Mental healthcare including diagnosis and treatment of, e.g. depression, anxiety and alcohol dependence is provided by PHC, while illicit drug use and severe opioid dependence are not conditions that are treated at PHCCs. In contrast to some places abroad, Swedish PHCCs do not offer OAT, which is only delivered at specialized public or private clinics. With 27 such clinics throughout the region, availability of OAT in Skåne is considered high in a national perspective. Besides the dispensing of buprenorphine or methadone, clinics are also required to provide psychological and psychosocial treatment, as well as control of blood borne infections.

### Participants

Nurses and physicians at PHCCs were invited to the study. Invitations were sent to the managers of 178 PHCCs in Skåne, based on the most comprehensive list of active PHCCs available. Out of the 178, 23 agreed to distribute the survey to their staff. Among the PHCC managers who declined participation, those who cited a reason all referred to time constraints and the high workloads of their staff. In the end, we received valid survey data from 13 PHCCs, employing a total of approximately 227 employed nurses (*n* = 108) and physicians (*n* = 119) at the time of the study, based on estimations on the part of the participating managers. All individual respondents received written information about the study, and their written consent to participate was collected. Prior to the study, ethics approval was obtained from the Swedish Ethical Review Authority (3 June 2021; file nr. 2021-02154).

### Procedures and data management

Participating PHCCs could choose if they wanted to be sent the survey questionnaires together with written information about the study by mail or have a researcher visiting them and introducing the survey to the staff. Eleven chose the former and two chose the latter. Surveys were completed at the PHCC, and either returned by mail or picked up by a researcher, at the discretion of clinic management. The reason for using a paper survey rather than an electronic one was to facilitate survey completion during staff meeting time at the PHCCs.

In the questionnaire, the respondents were asked for background data (sex, age, profession and specialty, years in the profession), and their attitudes toward PCPDU were assessed by using an adapted version of the Medical Condition Regard Scale (MCRS). Originally developed and validated (coefficient alpha = .87; test–retest reliability = .84) as a generic unidimensional scale to measure American medical students’ attitudes to patients with different medical conditions, MCRS assesses ‘the degree to which medical students find patients with a given medical condition to be enjoyable, treatable, and worthy of medical resources’ [29]. However, the scale has been used extensively to measure attitudes in a diverse range of not only students but also healthcare professionals – including PHC staff – in several national contexts [12,13,19,20].

The MCRS instrument consists of 11 items measured on a six-grade Likert scale, which aim to ‘capture biases, emotions, and expectations generated by medical condition descriptors’ [29]. Resulting scores range from 11 to 66, with higher scores reflecting more positive attitudes [29]. For this study, the instrument was translated to Swedish by the authors. Before being finalized, the translation was reviewed by a native-English speaking science editor who back-translated it to ensure accuracy. Two original items had to be adapted in order to work in the intended setting. First, an item that originally involved ‘getting up on call nights’ was modified to fit the PHC setting through a change of wording to ‘working inconvenient hours’. Second, an item concerning the coverage of health insurance plans, was deemed to work poorly in Sweden where healthcare coverage is universal. It was amended to instead concern the equitable right to healthcare. In the presentation of our results, we have kept the original English wording of all items that were left unaltered.

We measured attitudes to three types of patients with psychiatric conditions: patients with active illicit drug use, patients with OAT treated opioid dependence, and patients with depression. Depression was chosen to act as a reference condition, to make it possible to compare attitudes to drug use and OAT with a non-substance use psychiatric condition commonly treated in Swedish PHC.

### Analysis

The collected data were manually organized in SPSS Statistics Version 25 (IBM SPSS Statistics for Windows, Version 25.0, IBM Corp., Armonk, NY) by the authors and analyzed by using descriptive statistics.

We also used Mann–Whitney’s test to analyze potential associations between MCRS score and the covariates age, sex, profession and duration of professional experience. All covariates were dichotomized prior to analysis. *p* < .05 was considered statistically significant.

## Results

### Sample characteristics

A total of 89 PHC professionals responded to the survey, representing approximately 39% of those employed at the 13 PHCCs that participated. A majority (71%) of the respondents were female and the distribution between nurses and physicians was even (Table 1).

**Table 1.** Sample characteristics.

Characteristic	*n* (%)
Age	
18–34 years	19 (21)
35–44 years	32 (36)
45–54 years	21 (24)
≥55 years	17 (19)
Sex	
Female	63 (71)
Male	26 (29)
Profession	
Nurse (non-specialist)	25 (28)
Specialist nurse	20 (23)
Physician (non-specialist)	21 (24)
Specialist physician	23 (26)
Years of professional experience	
0–9 years	30 (34)
10–19 years	29 (33)
20≤ years	30 (34)

*N* = 89.

### MCRS scores

The median MCRS score was 44 for both patients with drug use and patients in OAT, and 51 for patients with depression (Table 2). Scores regarding patients with drug use and OAT patients displayed similar minimum, maximum and interquartile range values as well, while the scores regarding patients with depression displayed a higher minimum and much smaller interquartile range.

**Table 2.** Adapted Medical Condition Regard Scale scores.

MCRS score	Drug use	OAT	Depression
Median (min–max; IQR)	44 (23–63; 38–49)^a^	44 (24–63; 40–51)	51 (37–62; 47–55)
*n* (%) that agree with each statement			
1. I prefer not to work with patients like this	36 (49)	33 (37)	15 (17)
2. Patients like this irritate me	23 (26)	13 (15)	3 (3)
3. I enjoy giving extra time to patients like this	41 (46)^a^	41 (46)	65 (73)
4. Patients like this are particularly difficult for me to work with	47 (53)	34 (38)	19 (21)
5. Working with patients like this is satisfying	41 (46)	46 (52)	67 (75)
6. I feel especially compassionate toward patients like this	56 (63)	60 (67)	77 (87)
7. I would not mind working inconvenient hours to take care of patients like this	10 (11)	14 (16)	18 (20)
8. I can usually find something that helps patients like this feel better	60 (67)	63 (71)	85 (96)
9. There is little I can do to help patients like this	22 (25)	20 (23)	8 (9)
10. It is fair that healthcare services treat this type of patients equally as others who do not have the same problems.	79 (89)	80 (90)	88 (99)
11. Treating patients like this is a waste of medical dollars	6 (7)	4 (5)	5 (6)

IQR: interquartile range; OAT: opioid assisted treatment.

*N* = 89.

^a^ Missing *n* = 1.

Looking at responses to the individual MCRS questions, the same share of respondents agreed that they would enjoy spending extra time on both patients with active illicit drug use and patients in OAT. Roughly, the same share of respondents felt especially compassionate toward patients with active illicit drug use and patients in OAT, believed that both patient groups should enjoy equitable access to healthcare resources, and thought that there was little they as professionals could do to help such patients. Meanwhile, there were also some differences. A greater share of the respondents would prefer not to work with patients in active drug use, perceived them as irritating or particularly difficult to work with, while a slightly lower share agreed that they felt satisfaction working with them. In addition, slightly fewer indicated that they could usually help drug using patients feel better or that they would mind working inconvenient hours to care for them.

In comparison to depression, a substantially smaller share of respondents found it enjoyable to spend extra time on patients with either kind of drug related issue, experienced satisfaction working with them, felt especially compassionate toward them, or believed that they would usually find a way to make them feel better. Meanwhile, the proportions of respondents who would prefer not to work with depressed patients, found them to be irritating or agreed that there was little they could do to help them, were considerably lower than the corresponding numbers for patients with drug use related problems. The majority that supported equitable access to healthcare was even more pronounced for patients with depression than for the other patient types. Finally, the share of respondents who believed that treating any of the named patient groups would be a waste of healthcare resources was consistently small and similar across all the patient groups (5–7%).

### Factors associated with MCRS scores

We found no significant associations between the MCRS scores for either drug use or OAT, and age, sex, profession or duration of professional experience (Table 3).

**Table 3.** Factors associated with MCRS scores for patients with drug use/in OAT.

	Drug use^a^		OAT	
	Mean/median MCRS score	*p* Value	Mean/median MCRS score	*p* Value
Age		.65		.83
18–34 years	44/47		44/48	
≥35 years	44/44		45/44	
Sex		.79		.59
Female	44/44		44/44	
Male	43/46		45/47	
Profession		.54		.63
Nurse	44/45		44/44	
Physician	43/44		45/46	
Professional experience		.85		.45
0–19 years	44/44		45/45	
≥20 years	43/46		44/44	

MCRS: Medical Condition Regard Scale; OAT: opioid assisted treatment.

Mann–Whitney’s test. *N* = 89.

^a^ Missing *n* = 1.

## Discussion

This was the first study to measure attitudes to patients with current or previous drug use in Swedish PHC professionals, and – to our knowledge – the first study internationally to use the MCRS to measure attitudes to patients in OAT. We found that, among the survey respondents, attitudes to patients with active drug use or in OAT were very similar. While dichotomized data on the responses on the items suggested a somewhat more positive attitude to patients in OAT, the median score for the two conditions were identical. Compared to the mean score for depression, the scores for drug use and OAT treated opioid dependence were considerably lower, reflecting a more negative attitude to the former groups of patients than toward the latter. The fact that both drug use and OAT scored over the midpoint of the MCRS scale should not be interpreted as them being ‘above average’ or ‘adequate’, since the scale is known to suffer from ceiling effects, and to produce above-midpoint scores even for stigmatized conditions [29]. Mean and median MCRS scores for the drug related conditions showed little variation across genders, professions, age groups or groups of different work experience and we found no significant differences between different subsets of our sample.

The scores show a striking similarity to those measured for drug use and depression respectively in samples of healthcare workers in general in previous European studies [13,19], while the scores for the drug related conditions are higher than those previously found in PHC staff specifically [13,19,20]. Due to the limited sample size in our study, we could only speculate whether this finding might suggest that attitudes to patients presenting with drug related conditions are slightly more positive in the Swedish PHC setting studied here than in some other European countries.

Overall, patients with depression engendered more sympathetic attitudes, with respondents finding them more preferable and rewarding to work with, as well as easier to help, than PCPDU. While differences in MCRS scores between different conditions are not in themselves evidence of discriminatory behavior taking place in the clinic, the more negative attitudes toward PCPDU appear to reflect the experiences of negative reception in healthcare found in the group in previous studies [10]. However, regardless of their views in other matters, few respondents believed either that PCPDU should receive healthcare on other terms than patients with other problems, or that providing them with healthcare should be considered as a waste of resources. This testifies to a relatively strong support of equal treatment as an ideal and of the formal rights of patients in our sample, which reflects central norms inscribed in Swedish health services legislation. Meanwhile, it indicates that targeted educational interventions to increase PHC staff competence on addiction issues could well have a beneficial effect, as it could make it easier for PHC professionals to identify feasible ways to help PCPDU and make them feel more comfortable working with such patients. Interestingly, attitudes toward patients in active drug use and those in treatment were notably similar, rendering the same median MCRS score. Previous findings have suggested that OAT patients continue to face poor treatment from healthcare workers, even though they are enrolled in treatment programs and no longer supposed to use drugs illicitly [10,21]. There are several possible explanations to the similarities. They could be the result of a lack of discrimination between the two conditions, e.g. because of an ignorance among PHC professionals of the aims and functions of OAT. However, it could also be the result of so-called intervention stigma, i.e. stigma carried by OAT in itself, divorced from the condition stigma tied to drug dependence per se [22]. Finally, since our definition of active drug use included use of the full spectrum of illicit drugs, from cannabis to stimulants and opioids, it is possible that participants imagined this group of patients as having less severe problems, in comparison to OAT treated opioid dependence, a condition which could possibly elicit associations to more severe substance use problems [17].

This study has limitations. The sample is relatively small and, although the response frequency was fair at the sites where recruitment took place, as a result of the low participation rate among the invited PHCCs, the survey reached only a small minority of the PHC physicians and nurses employed in the studied region. The remarkable similarities between the average MCRS scores found for drug related conditions here and in previous studies might be taken to indicate that our sample was not extreme, but the fact that we cannot guarantee its representativity calls for caution in drawing more general conclusions.

In conclusion, the results indicate widespread negative attitudes to PCPDU among the study respondents, and that attitudes to patients with illicit drug use and patients in OAT are largely similar. This has potentially severe negative consequences for PCPDU in need of PHC, and thus clear implications for health equity in the clinic. However, there is a need for larger scale studies, preferably based on randomized samples of Swedish PHC professionals, to investigate whether the results in our study can be reproduced. Meanwhile, qualitative research methods could be employed to gain a deeper and much needed understanding of the similarities in attitudes taken to patients with current illicit drug use and patients in OAT.
